# Contributions of two cytosolic glutamine synthetase isozymes to ammonium assimilation in Arabidopsis roots

**DOI:** 10.1093/jxb/erw454

**Published:** 2016-12-22

**Authors:** Noriyuki Konishi, Keiki Ishiyama, Marcel Pascal Beier, Eri Inoue, Keiichi Kanno, Tomoyuki Yamaya, Hideki Takahashi, Soichi Kojima

**Affiliations:** 1 Graduate School of Agricultural Science, Tohoku University, 1-1 Tsutsumidori-Amamiyamachi, Sendai, 9818555, Japan; 2 RIKEN Plant Science Center, Yokohama, 2300045, Japan; 3 Department of Biochemistry and Molecular Biology, Michigan State University, East Lansing, MI 48824, USA

**Keywords:** Ammonium, *Arabidopsis*, glutamine, GS1, nitrogen, root.

## Abstract

Glutamine synthetase (GS) catalyzes a reaction that incorporates ammonium into glutamate and yields glutamine in the cytosol and chloroplasts. Although the enzymatic characteristics of the GS1 isozymes are well known, their physiological functions in ammonium assimilation and regulation in roots remain unclear. In this study we show evidence that two cytosolic GS1 isozymes (GLN1;2 and GLN1;3) contribute to ammonium assimilation in Arabidopsis roots. Arabidopsis T-DNA insertion lines for *GLN1;2* and *GLN1;3* (i.e. *gln1;2* and *gln1;3* single-mutants), the *gln1;2:gln1;3* double-mutant, and the wild-type accession (Col-0) were grown in hydroponic culture with variable concentrations of ammonium to compare their growth, and their content of nitrogen, carbon, ammonium, and amino acids. *GLN1;2* and *GLN1;3* promoter-dependent green fluorescent protein was observed under conditions with or without ammonium supply. Loss of *GLN1;2* caused significant suppression of plant growth and glutamine biosynthesis under ammonium-replete conditions. In contrast, loss of *GLN1;3* caused slight defects in growth and Gln biosynthesis that were only visible based on a comparison of the *gln1;2* single- and *gln1;2:gln1;3* double-mutants. *GLN1;2*, being the most abundantly expressed GS1 isozyme, markedly increased following ammonium supply and its promoter activity was localized at the cortex and epidermis, while *GLN1;3* showed only low expression at the pericycle, suggesting their different physiological contributions to ammonium assimilation in roots. The *GLN1;2* promoter-deletion analysis identified regulatory sequences required for controlling ammonium-responsive gene expression of *GLN1;2* in Arabidopsis roots. These results shed light on *GLN1* isozyme-specific regulatory mechanisms in Arabidopsis that allow adaptation to an ammonium-replete environment.

## Highlight

Temporal and spatial distributions of GLN1;2 and GLN1;3, the two low-affinity cytosolic glutamine synthetase isoforms, determine their contributions to ammonium assimilation in *Arabidopsis* roots.

## Introduction

Ammonium and nitrate are inorganic nitrogen forms used in plant growth (von Wirén *et al.*, 2000). Plants may preferentially take up ammonium for energy conservation when both nitrate and ammonium are present (Sasakawa and Yamamoto, 1978; Gazzarrini *et al.*, 1999; Gu *et al.*, 2013). However, given that excessive ammonium supply may inhibit plant growth (von Wirén *et al.*, 2000; Britto and Kronzucker, 2002; Hachiya *et al.*, 2012), ammonium must be quickly assimilated into glutamine (Andrews *et al.*, 2013; Yamaya and Kusano, 2014). The glutamine synthetase/glutamate synthase (GS/GOGAT) cycle is the key step in ammonium assimilation in higher plants (Tobin and Yamaya, 2001; Lea and Azevedo, 2007). Glutamine synthetase (GS or GLN) catalyzes a reaction that incorporates ammonium into glutamate and generates glutamine as a product in an adenosine triphosphate (ATP)-dependent manner (Tobin and Yamaya, 2001). Glutamate synthase (also termed glutamate 2-oxoglutarate aminotransferase, GOGAT) transfers the amine group in the amide side chain of glutamine to 2-oxoglutarate, yielding two molecules of glutamate; one molecule serves as a substrate for GS, whilst the other is used for transport, storage, or further metabolism (Tobin and Yamaya, 2001). GS is categorized into two groups: (1) the cytosol-localized GS1 group, and (2) the GS2 group localized mainly in the chloroplasts (Swarbreck *et al.*, 2011). In the Arabidopsis genome, a single *GLN2* gene and five *GLN1* genes are encoded. A barley mutant lacking functional GS2 does not grow normally under ambient-CO_2_ conditions; however, this growth defect is rescued under high CO_2_ conditions (Blackwell *et al.*, 1988). Thus, it has been suggested that GS2 could assimilate the ammonium derived from photorespiration (Wallsgrove *et al.*, 1987), whereas GS1 isozymes assimilate non-photorespiratory ammonium (Tobin and Yamaya, 2001). In addition to primary uptake and photorespiration, ammonium can originate in several metabolic processes, including nitrate reduction, phenylpropanoid metabolism, degradation of transported amides, and protein catabolism (Schjoerring *et al.*, 2002; Li *et al.*, 2014). Four GS1 isozymes of Arabidopsis, encoded by *GLN1;1*, *GLN1;2*, *GLN1;3*, and *GLN1;4*, have been identified to have different enzymatic characteristics when they are expressed in *E. coli* (Ishiyama *et al.*, 2004). Individual GS1 isoenzymes may share assimilatory functions for the ammonium originating in non-photorespiration (Yamaya and Kusano, 2014). Analysis of mutants lacking a specific GS1 isozyme suggests that GS1 functions in non-photorespiratory ammonium assimilation in monocotyledonous crop plants, such as rice (Tabuchi *et al.*, 2005; Funayama *et al.*, 2013) and maize (Martin *et al.*, 2006; Cañas *et al.*, 2010). Phylogenetic analysis further suggests key differences between crop and Arabidopsis GS1 amino acid sequences (Thomsen *et al.*, 2014), and the isogene-specific physiological functions of GS1 in Arabidopsis have been only partially documented or studied, with a focus on their roles in nitrogen remobilization in aerial organs based on their predominant expression being found in vascular tissues (Thomsen *et al.*, 2014; Guan *et al.*, 2015).

Three previous studies have reported on the physiological functions of GS1 isozymes in Arabidopsis using reverse-genetic approaches (Lothier *et al.*, 2011; Guan *et al.*, 2015, 2016). *GLN1;2* is essential for nitrogen assimilation and ammonium detoxification (Lothier *et al.*, 2011; Guan *et al.*, 2016). *GLN1;2* promoter activity is localized mainly in the minor veins of leaves and flowers, and the GLN1;2 protein is localized in companion cells (Lothier *et al.*, 2011). Transfer DNA (T-DNA) insertion lines for *GLN1;2* showed a decrease in GS activity and rosette biomass compared with the wild-type under nitrate-sufficient conditions; however, no significant difference in nitrogen remobilization was found. When ammonium was supplied as the sole nitrogen source after pre-culture in nitrate-sufficient conditions, *GLN1;2* insertion lines developed root hairs and had reduced rosette sizes (Lothier *et al.*, 2011). Guan *et al.* (2015) reported that *GLN1;2* plays an important role in nitrogen remobilization. Both the single T-DNA insertion line for *GLN1;2* and the double-insertion line for *GLN1;1* and *GLN1;2* showed a decrease in seed yield, whereas the single-insertion line for *GLN1;1* showed a yield comparable to the wild-type. The *GLN1;2* promoter-dependent green fluorescent protein (GFP) showed fluorescence localized in the vascular cells of roots, petals, and stamens (Guan *et al.*, 2015). A more recent study showed that GLN1;2 is the main isozyme contributing to shoot GS1 activity in the vegetative growth stage and that it can be up-regulated to relieve ammonium toxicity (Guan *et al.*, 2016). However, there remains a need for an efficient method that minimizes the nitrate used in the nutrient solution.

The enzymatic characteristics of recombinant GLN1;2 and GLN1;3 suggest that these two GS1 isozymes with low substrate affinities may contribute to ammonium assimilation in Arabidopsis under ammonium-replete conditions (Ishiyama *et al.*, 2004). However, the role-sharing of GLN1;2 and GLN1;3 in ammonium-supplied roots remains to be elucidated. The present study provides evidence that *GLN1;2* and *GLN1;3* are necessary for ammonium assimilation in Arabidopsis roots, particularly in roots exposed to high concentrations of ammonium supply, based on results obtained through a reverse genetic approach using T-DNA insertion mutants and promoter-GFP lines reporting their differential physiological functions and spatio-temporal regulation. The finding of ammonium-responsive regulatory sequences in the *GLN1;2* gene promoter region further implies a distinct contribution of the GLN1;2 isozyme to ammonium assimilation in roots under ammonium-replete conditions.

## Materials and methods

### *Isolation of T-DNA insertion lines for* GLN1;2 *and* GLN1;3

Arabidopsis (*Arabidopsis thaliana*) accession line Columbia (Col-0) was used as the wild-type (WT). The following T-DNA insertion lines in the Col-0 genetic background were used: *gln1;2-1* (At1g66200; SALK_145235), *gln1;2-2* (SALK_102291), *gln1;3-1* (At3g17820; SALK_002524), *gln1;3-2* (SALK_038156), and *gln1;3-3* (SALK_148604C). T-DNA insertion lines were obtained from the SALK institute, self-fertilized, and selected for T-DNA homozygous plants. The T-DNA positions were determined by PCR using primers for T-DNA, *T-DNA LB-01* (5′-CCAGTACATTAAAAACGTCCG CAATGTGTT-3′) and *T-DNA RB-01* (5′-CCGAATACAGTGATCCG TGCCGCCCTG-3′); for the *GLN1;2* gene, *GLN1;2F* (5′-ATGAGTCT TCTTGCAGATCTTGTTA-3′) and *GLN1;2R* (5′-GGTTTC AATAAAGGTCAAACAAACAGA-3′); and for the *GLN1;3* gene, *GLN1;3F* (5′-ATGTCTCTGCTCTCAGATCTCGTTA-3′) and *GLN1;3R* (5′-TCAACCGAGTATGGTCGTCTCAGCG-3′)

Two T-DNA insertion lines, *gln1;2-1* and *gln1;3-1*, were crossed, and the double-insertion line, *gln1;2:gln1;3* was isolated.

### Hydroponic culture

Three to five Arabidopsis seeds were germinated on water-moistened rock wool for 4 d in the dark, and single seedlings were selected. Plants were transferred to a hydroponic nutrient solution described by Loqué *et al.* (2006) with modifications. The modified hydroponic solution was buffered with 5 mM 2-(N-morpholino) ethanesulfonic acid (MES) adjusted to a pH of 5.8 with KOH, and 2 mM NH_4_NO_3_ was removed to be replaced with 10 μM KNO_3_ and various concentrations of NH_4_Cl, given that a small amount of nitrate alleviates the detrimental effects of pure ammonium nutrition (Krouk *et al.*, 2006; Garnica *et al.*, 2010). The nutrient solution was always buffered with MES. Plants were grown in three sizes of pots in pre-culture because of space limitations. First, at ammonium concentrations of 0.1, 0.3, 1, 2, 3, 5, and 10 mM, 18 WT plants were grown in a 0.8-l plastic container filled with 0.7 l of nutrient solution. Second, at ammonium concentrations of 0.1, 0.3, 0.5, and 1 mM, 220 plants (44 plants per line, five genotypes) were grown in a 2-l plastic container filled with 2 l of nutrient solution. Third, at ammonium concentrations of 0.1 or 3 mM, 120 plants (17 plants per line, seven genotypes) were grown in a 5.9-l plastic container filled with 5 l of nutrient solution. All plastic containers were purchased from Sanko Co., Ltd, (Tokyo, Japan).

Six to eight plants from the pre-culture were then transferred at 21 to 25 d after sowing to a black acrylic resin plate (0.11 × 0.15 m, 5 mm thick) with nine holes. A 0.8-l plastic container was filled with 0.7 l of hydroponic solution and covered with the resin plate. The hydroponic solution was exchanged twice weekly. Plants were grown in a climate chamber (Biotron LPH-350S, Nippon Medical and Chemical Instruments Co., Ltd, Tokyo, Japan; 10/14 h light/dark, 22 °C, 60% humidity, and 160 μmol m^–2^ s^–1^ total light intensity). Each plastic container was aerated by pumping. Roots and shoots were harvested separately 6 weeks after sowing. Roots were washed in 1 mM CaSO_4_ solution for 1 min before harvest.

Samples were collected in envelopes or 2-ml safe-lock tubes (Eppendorf Co., Ltd, Tokyo, Japan). The samples were dried in an oven at 80 ºC for several days. A zirconia bead was added to the 2-ml safe-lock tubes for milling samples. The hydroponic solution was renewed 3 d before harvesting. The harvesting began 3 h into the light period.

Samples were frozen in liquid nitrogen immediately after measurement of the fresh weight using CPA324S electronic balance (Sartorius Japan K.K., Tokyo, Japan). Samples for quantitative real-time polymerase chain reaction (qPCR) and amino acid measurements were maintained at −80 °C. Samples for dry weight measurement were dried in an oven at 80 °C for 4–7 d and weighed with an electronic balance (XS Analytical Balances, Mettler-Toledo International Inc, Columbus, USA). Experiments were repeated at least twice obtaining similar results, and representative values from one experiment are shown in the figures.

### Cellular localization of GLN1;2 and GLN1;3 promoter activities

The *GLN1;2* upstream region was amplified from Col-0 genomic DNA by PCR. KOD -Plus- DNA polymerase (Toyobo Co., Ltd, Osaka, Japan) was used in the PCR with gene-specific primers, *GLN1;2P5697L_F*: (5′-GGGATCCGATGTAGATGATTAAAGAT ATATAACTA-3′) and *GLN1;2P2501L_F*: (5′-CGGATCC ATTTTAGCAAGAGACCATCCACACTAAC-3′), paired with a reverse primer, *GLN1;2P_R*: (5′-GCCATGGGGTTGCAA GAAGAAACAAGAAGATTGAA-3′). The region upstream of the *GLN1;2* start codon was tagged with restriction sites for *Bam*HI (GGATCC) and *Nco*I (CCATGG). The entire *GLN1;2* promoter region at different lengths (5697 bp or 2501 bp) was then fused with enhanced green fluorescent protein (GFP; Takara Bio Inc.) using the *Nco*I restriction site designed in the *GLN1;2P_R* primer. The region upstream of the 2501-bp *GLN1;2* promoter was amplified from genomic DNA by PCR with gene-specific primers, *GLN1;2P5372L_F*: (5′-GAAGCTTCATTTAAGTTTTGTACGACATCTAATT-3′), *GLN1;2P3822L_F*: (5′-GAAGCTTGCGACAGAAAAAAAG AAAACAAGACAT-3′), *GLN1;2P3624L_F*: (5′-GAAGCTTTT TTTTTTTTTAGTTTGTCTTTTTTTTT-3′), *GLN1;2P3604L_F*: (5′-GAAGCTTGTCTTTTTTTTTTACCGTCAACTCTTAC-3′), *GLN1;2P3563L_F*: (5′-GAAGCTTTTCTTAACTGT ATGACACCA TTGCTTAC-3′), *GLN1;2P3522L_F*: (5′-GAAGCTTCTGGT AAATTATATTACCATTTCTATAA-3′), *GLN1;2P3430L_F*: (5′-GAAGCTTGGCATCTACACTTCATAAAGTGTCG ACATC-3′), paired with a reverse primer, *GLN1;2P_R_02*: (5′-GGGAT CCTAGACTGCGTGAGAATGTAAAAATGTAA-3′). The region was tagged with restriction sites for *Hin*dIII (AAGCTT) and *Bam*HI. The partial fragment of the *GLN1;2* promoter region at different lengths was then fused with the upper region of the 2501-bp GLN1;2 promoter using the *Bam*HI restriction site designed in the *GLN1;2P_R_02* primer. The entire *GLN1;2* promoter region at different lengths (5372, 3822, 3624, 3604, 3563, 3522, or 3430 bp) was then fused with enhanced GFP (Takara Bio Inc.). The *GLN1;2* promoter-GFP fragment was ligated to a pBI101 (Clontech, Palo Alto, CA) -based binary vector, as previously described by Ishiyama *et al.* (2004). The binary plasmids were transferred to *Agrobacterium tumefaciens* GV3101, and Arabidopsis plants were transformed according to the floral dip protocol (Clough and Bent, 1998). The *GLN1;3* promoter-GFP lines originated from our previous study (Ishiyama *et al.*, 2004).

Plants were grown in hydroponic culture or on vertical agar plates. In the hydroponic culture, plants were grown for 6 weeks in nutrient solution containing 0.1 or 3 mM ammonium and 10 µM nitrate as nitrogen sources. Laser-scanning confocal microscopy was performed with a Nikon C1si System. A CFI Plan Fluor 20× (numerical aperture 0.5; Nikon) or a CFI Plan Apo Lambda 40× (numerical aperture 0.95; Nikon) was used as the objective lens. GFP was excited with the 488-nm line of a multi-argon ion laser. The fluorescence spectra between 500 and 530 nm were obtained with the spectral detector of the Nikon C1si System. Plants that were cultured on vertical agar plates were placed in a growth cabinet at 22 °C with 60% relative humidity under 16/8 h light/dark cycles, as previously reported (Ishiyama *et al.*, 2004). The light intensity used was 40 μmol m^–2^ s^–1^. Three steps controlled the plant nitrogen nutrition: (1) plants were grown on MGRL agar medium (Fujiwara *et al.*, 1992) containing 7 mM nitrate as the major nitrogen source for 14 d; (2) plants were transferred to nitrogen-free MGRL medium and pre-cultured for 3 d to facilitate nitrogen starvation; and (3) plants were then transferred again to N-free MGRL medium either supplemented with 10 mM ammonium as the sole nitrogen source or containing no nitrogen source and incubated for 24 h for confocal microscopy or 9 h for qPCR analysis of GFP expression. Plants were all cultured under sterile conditions. Confocal laser scanning microscopic analysis was performed using a BX61 microscope equipped with a FV500 with a 505–525 nm band pass filter (Olympus, Tokyo, Japan) for detection, as descried previously (Ishiyama *et al.*, 2004). Images were processed in Adobe Photoshop.

### Quantitative real-time PCR analysis and reverse transcription (RT)-PCR analysis

Messenger RNA (mRNA) was quantified by quantitative PCR (qPCR) as previously described (Konishi *et al.*, 2014). Plants were grown hydroponically in nutrient solution with 0.1, 1, or 3 mM NH_4_Cl and 10 μM KNO_3_ for 6 weeks. Total RNA was extracted with an RNeasy Plant Mini Kit (Qiagen, K. K., Tokyo, Japan). Absorbances at 260 nm and 280 nm were measured with a NanoDrop 1000 spectrophotometer (NanoDrop, LMS Co., Ltd Tokyo, Japan) to quantify and characterize the extracted RNA. RT and DNase treatments were performed using a PrimeScript^®^ RT reagent Kit with genomic (g) DNA Eraser (Takara Bio Inc., Otsu, Japan) with 500 ng of total RNA in a 20 μL final volume, according to the manufacturer’s instructions. The products were diluted five times with RNase-free water and used as a template. PCR reactions were performed on a Light Cycler^®^ 480 (Roche Diagnostics K.K., Tokyo, Japan), according to the following program: 10 s at 95 °C, followed by 50 cycles of 95 °C for 5 s, and 60 °C or 65 °C for 34 s. SYBR Premix Ex Taq™ II (Takara Bio Inc.), 2 μl complementary (c)DNA sample as a template, and 0.4 μM of each gene-specific primer were reacted. Gene-specific primers for *GLN1;1*, *1;2*, *1;4*, *2*, and *ubiquitin2* (*UBQ2*; GenBank J05508) were prepared following the method of Ishiyama *et al.* (2004). *GLN1;3*-specific primers were *GLN1;3-RTF* (5′-TCCAACCAACAAGAGGCACAAC-3′) and *GLN1;3-RTR* (5′-ACCAGAACTAATACCCTCAACA-3′). *GFP* specific primers were 204F (5′-AGTGCTTCAGCCGCTACCC-3′) and 345R (5′-CCCTCGAACTTCACCTCGG-3′). Serial dilutions of plasmid were used as standards. Data were acquired and analyzed using the Light Cycler 480 Software version 1.2 (Roche Diagnostics K.K.). The dissociation curve confirmed a single PCR product. Water was used as a non-template control. The signal intensity was standardized to *UBQ2*. Three independent samples were quantified. The fold-change in gene expression relative to that of the WT at 1 mM ammonium was determined on the basis of crossing-point (CP) values (Pfaffl, 2001). RT-PCR primers for *GLN1;2*-specific primers were *Gln1;2RF and NK124* (5′-CGGATCATCCTTTC AAGGGTTCCAGAGGAG-3′), for *GLN1;3*-specific primers they were *NK145* (5′-ATGTCTCTGCTCTCAGATCTCGTTA-3′) and *NK146* (5′-TCAACCGAGTATGGTCGTCTCAGCG-3′), and *UBQ2*-specific primers were prepared following the method of Ishiyama *et al.* (2004).

### Nitrogen and carbon content

Plants were grown in a nutrient solution containing either 0.1 or 1 mM NH_4_Cl as the major nitrogen source for 6 weeks. Plant samples were dried and powdered with a Tissue Lyser II (Qiagen, K. K.) at 20 Hz for at least 15 min. Samples were weighed with an ultra-microbalance (UMX2, Mettler Toledo International Inc., Tokyo, Japan) in tin capsules. The weights of samples were always between 1.000 and 1.050 mg. Nitrogen and carbon were determined using an elemental analyzer (Flash2000, Thermo Fisher Scientific K. K., Yokohama, Japan).

Uptake efficiency (UpE) and usage index (UI) (Good *et al.*, 2004) were calculated to evaluate nutrient use efficiency in the WT and *GLN1* insertion lines. UI is an index for the efficiency with which the N absorbed is utilized to produce biomass (Siddiqi and Glass, 1981). UpE is an index for the efficiency of uptake (Moll *et al.*, 1982). Experiments were repeated at least twice with similar results, and representative values from one experiment are shown.

### Measurement of free amino acids and ammonium

Plant samples were frozen in liquid nitrogen and then milled using the Tissue Lyser II at 23 Hz for 1 min. Samples were suspended in 10 mM HCl and mixed in the Tissue Lyser II at 20 Hz for 2 min. After centrifugation at 20 500 *g* for 15 min at room temperature, the supernatant was transferred to an Amicon^®^ Ultra 3K filter cup (Millipore, Bedford, MA) on a 2-ml tube and centrifuged again at 20 500 *g* for 30 min at room temperature. Amide residues of both amino acids and ammonium were labeled using the AccQ-Tag Ultra Derivatization Kit (Nihon Waters K. K., Tokyo, Japan), as previously described by Konishi *et al.* (2014). Labeled samples were separated and analyzed on an ACQUITY Ultra Performance Liquid Chromatograph (UPLC) H-Class (Nihon Waters K. K.). Experiments were repeated at least twice with similar results and representative values from one experiment are shown.

### Xylem sap preparation

Plants were grown hydroponically in nutrient solution (Loqué *et al.*, 2006) for 42 d and transferred to a nutrient solution without nitrogen for 3 d. Plants were transferred again to the solution containing 0.1 or 3 mM NH_4_Cl and 10 μM KNO_3_ and the hypocotyls were excised with a razor (Feather Safety Razor Co., Ltd, Osaka, Japan) to collect xylem sap. Xylem sap was collected by harvesting the leaching solution from a cross-section at 24 h after plant transfer. The xylem sap was collected for 30 min after excision. Ammonium supply always started at 3 h into the light period. Experiments were repeated at least twice with similar results, and representative values from one experiment are shown.

### Statistics

All data sets were analyzed using Microsoft Excel add-in software (Social Survey Research Information Co., Ltd, Tokyo, Japan).

## Results

### Dose-dependent effects of ammonium on Arabidopsis growth under low-nitrate conditions in hydroponic culture

**Fig. 1.**
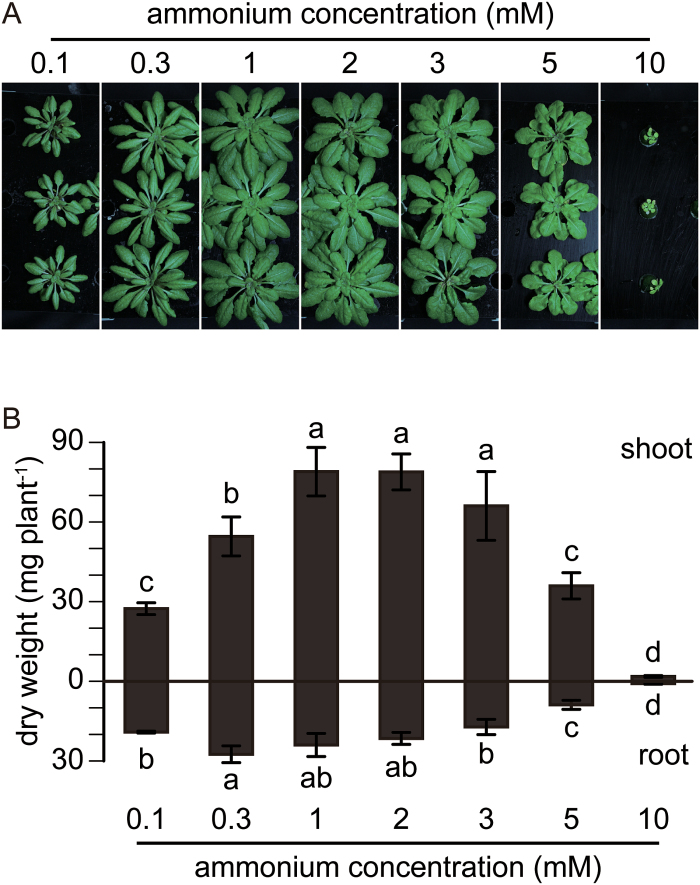
Growth of the wild-type (WT) under increasing concentrations of ammonium. (A) Growth of the WT in hydroponic solutions containing 0.1, 0.3, 1, 2, 3, 5, or 10 mM NH_4_Cl as the major nitrogen source, supplemented with 10 μM nitrate for 6 weeks. (B) Shoot and root dry weights of the same plants as in (A). Data are means ±SD (*n* = 4). One-way ANOVA followed by Bonferroni tests were used, and significant differences at *P*<0.05 within each group are indicated by different letters. (This figure is available in colour at *JXB* online.)

We evaluated the growth of *A. thaliana* Col-0 (WT) in a nutrient solution containing 10 μM KNO_3_, and supplemented with 0.1, 0.3, 1, 2, 3, 5, or 10 mM NH_4_Cl. Figure 1A shows the phenotype of the WT plants after 6 weeks in a hydroponic culture. As can be seen, WT shoots showed maximal growth under 1–2 mM NH_4_Cl. However, growth was decreased when the concentration of NH_4_Cl in the nutrient solution was >2 mM. The shoot and root dry weights of the WT plants tended to decrease at 3 mM, and were reduced by half at 5 mM compared with those at 1 mM Fig. 1B. Growth was even more strongly inhibited at 10 mM.

### GLN1;2 is the main isoform that assimilates ammonium over a wide range of growth concentrations

**Fig. 2.**
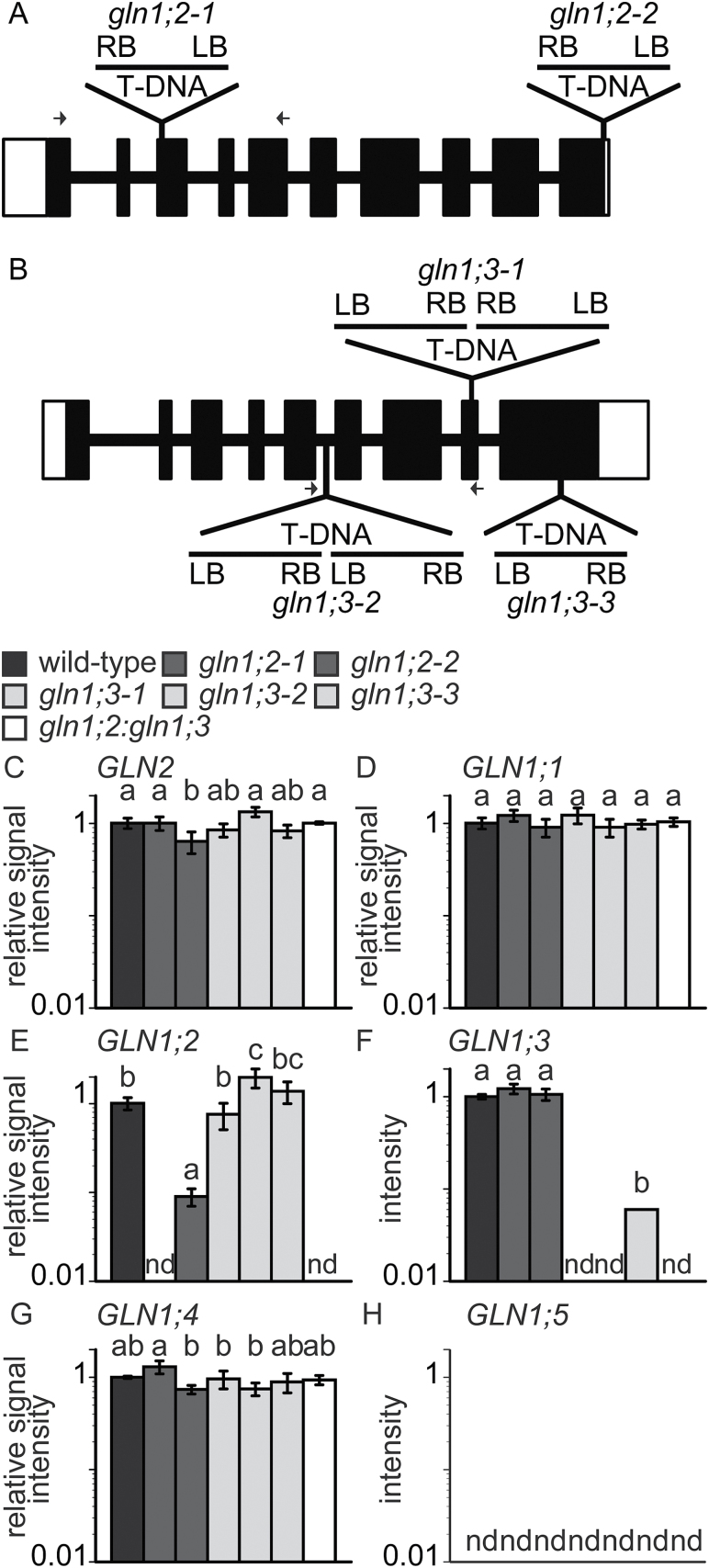
Isolation of T-DNA insertion lines for *GLN1;2* and *GLN1;3*. (A) The positions of the T-DNA insertions in *gln1;2-1* and *gln1;2-2*. (B) The positions of the T-DNA insertions in *gln1;3-1*, *gln1;3-2*, and *gln1;3-3*. Exons are indicated as black boxed regions, lines represent introns, and white boxes correspond to 5′- and 3′-untranslated sequences. Arrows indicate the positions of gene-specific primers used for quantitative polymerase chain reaction (qPCR). (C–G) Quantitative real-time PCR analysis of root RNA from the WT, *gln1;2-1*, *gln1;2-2*, *gln1;3-1*, *gln1;3-2*, *gln1;3-3*, and *gln1;2:gln1;3* (as indicated in the key) using gene-specific primer sets for *GLN2* (C), *GLN1;1* (D), *GLN1;2* (E), *GLN1;3* (F), *GLN1;4* (G), and *GLN1;5* (H). Plants were grown in hydroponic culture with 1 mM ammonium and 10 μM nitrate for 6 weeks. *UBQ2* was used to standardize the signal intensity. Data are means ±SD (*n* = 3). One-way ANOVA followed by Bonferroni tests were used, and significant differences at *P*<0.05 within each group are indicated by different letters. The fold-change in gene expression relative to that of the WT at 1 mM ammonium was determined on the basis of crossing-point (CP) values (Pfaffl, 2001).

Two T-DNA insertion lines for *GLN1;2* (Fig. 2A) and those for *GLN1;3* (Fig. 2B) were isolated from the WT. One of the T-DNA insertion lines, SALK_102291, was identical to that of a previous study by Lothier *et al.* (2011), whilst SALK_148604 was identical to that of a study by Dragićević *et al.* (2014). qPCR analysis showed that *GLN1;2* mRNA was not detectable in *gln1;2-1*, whereas it was slightly expressed in *gln1;2-2* (Fig. 2E). However, RT-PCR showed no visible *GLN1;2* expression in either T-DNA insertion line (data not shown). The expression of the other GS isozymes, *GLN1;1* (Fig. 2D), *GLN1;3* (Fig. 2F), *GLN1;4* (Fig. 2G), and *GLN2* (Fig. 2C) appeared unchanged in the T-DNA insertion lines for *GLN1;2*. *GLN1;5* was not detectable in the roots (Fig. 2H).

**Fig. 3.**
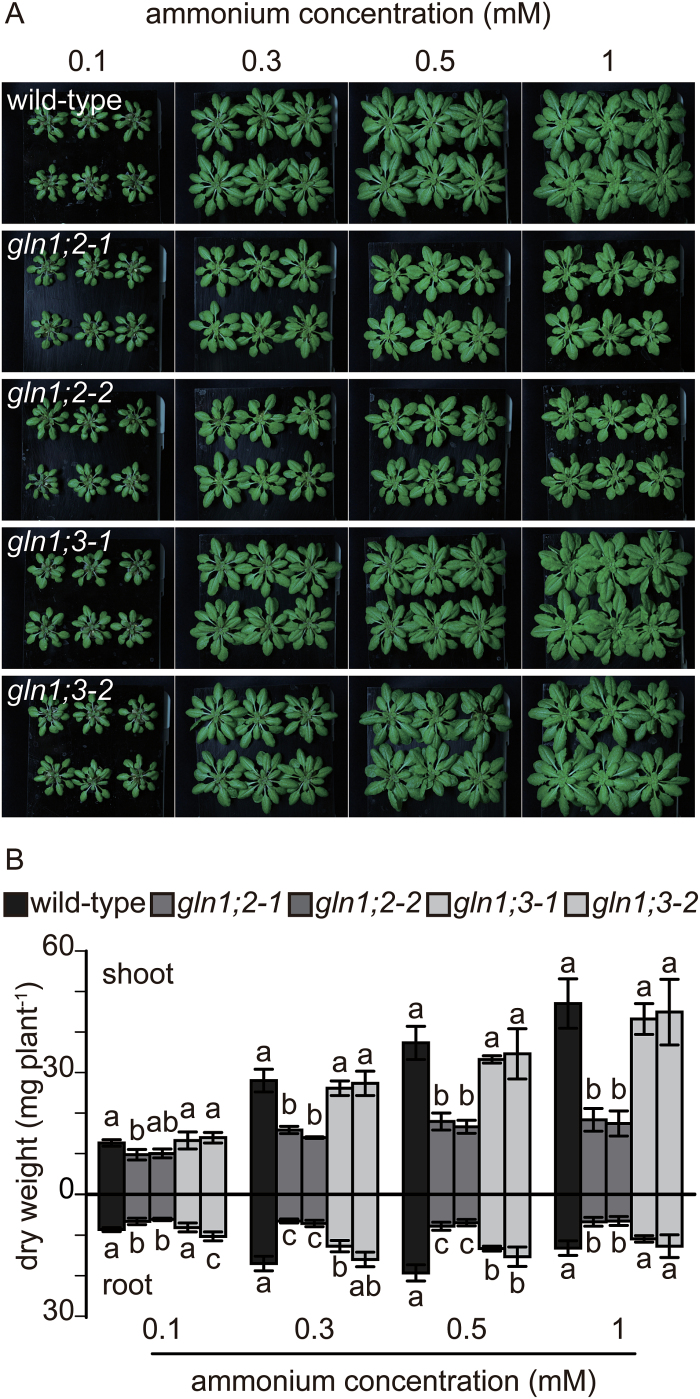
Growth of the wild-type (WT) and T-DNA insertion lines for *GLN1;2* and *GLN1;3* under low nitrate supply, and the effects of varied ammonium supply in the nutrient solution. (A) Phenotype of the WT and insertion lines for *GLN1;2* and *GLN1;3*. (B) Shoot and root dry weights of the WT, and the *GLN1;2* and *GLN1;3* insertion lines (as indicated in the key). Plants were grown for 6 weeks in nutrient solutions containing 0.1, 0.3, 0.5, or 1 mM ammonium, and 10 μM nitrate as the nitrogen source. Data are means ±SD (*n* = 6). One-way ANOVA followed by Bonferroni tests were used, and significant differences at *P*<0.05 within each group are indicated by different letters. (This figure is available in colour at *JXB* online.)

Figure 3 shows the different contributions of *GLN1;2* and *GLN1;3* to ammonium nutrition. The *GLN1;2* insertion lines showed a marked reduction in dry weight compared with the WT. In addition, supplying ammonium led to a dose-dependent reduction in dry weight of the *GLN1;2* insertion lines, whereas the *GLN1;3* insertion lines showed no reduction. The *GLN1;2* insertion lines showed a 60% reduction in dry weight at 1 mM ammonium, but only a 25% reduction at 0.1 mM (Fig. 3A, B). Conversely, there were no significant differences between the WT and *gln1;2* in the nutrient solution containing either 1 or 10 mM nitrate (Supplementary Fig. S2 at *JXB* online).

**Fig. 4.**
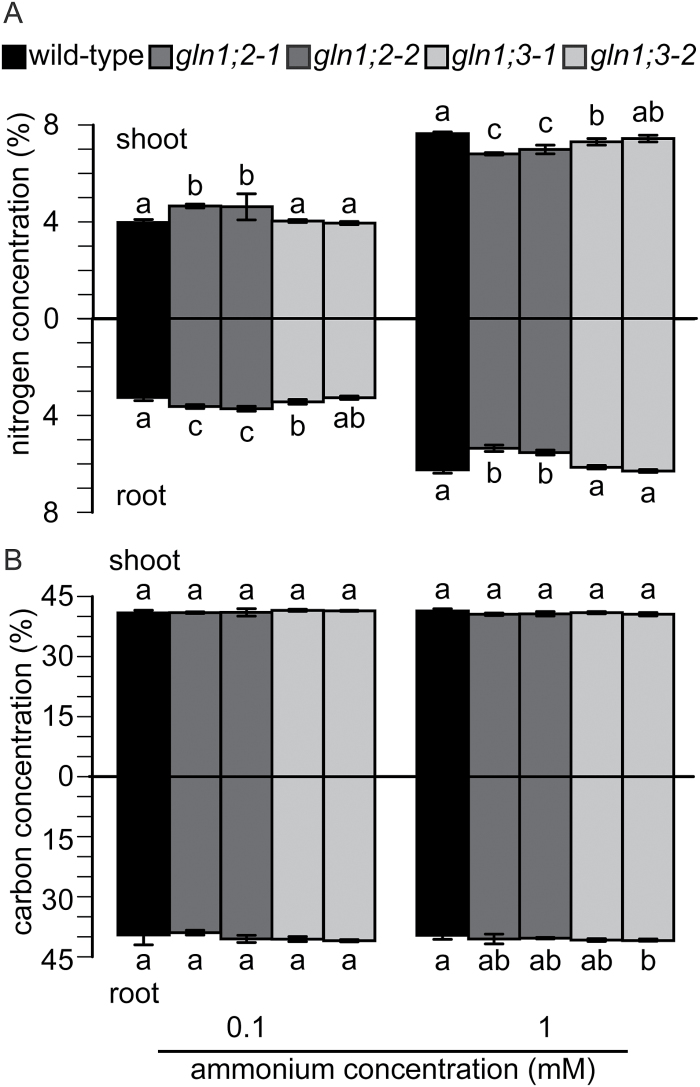
Total nitrogen and carbon contents in roots and shoots of the wild-type (WT) and the *GLN1;2*, and *GLN1;3* insertion lines. (A) Total nitrogen contents and (B) total carbon contents in roots and shoots of the WT and the *GLN1;2* and *GLN1;3* insertion lines (as indicated in the key). Plants were grown hydroponically, and supplemented with either 0.1 or 1 mM ammonium for 6 weeks. Data are means ±SD (*n* = 6). One-way ANOVA followed by Bonferroni tests were used, and significant differences at *P*<0.05 within each group are indicated by different letters.

Figure 4 shows nitrogen and carbon concentrations in the shoots and roots of the *GLN1* insertion lines. Carbon concentration in the shoots and roots ranged from 35 to 40%, and there were no significant differences with the WT (Fig. 4B). The total nitrogen concentrations in the WT ranged from 3 to 4% at 0.1 mM ammonium, and increased to 6–8% under 1 mM ammonium (Fig. 4A). Nitrogen concentrations in the *GLN1;2* insertion lines were significantly higher than those in the WT under 0.1 mM ammonium, and were lower under 1 mM (Fig. 4A). The *GLN1;3* insertion lines showed no clear changes in nitrogen concentration under either 0.1 or 1 mM ammonium in comparison with the WT (Fig. 4A).

**Fig. 5.**
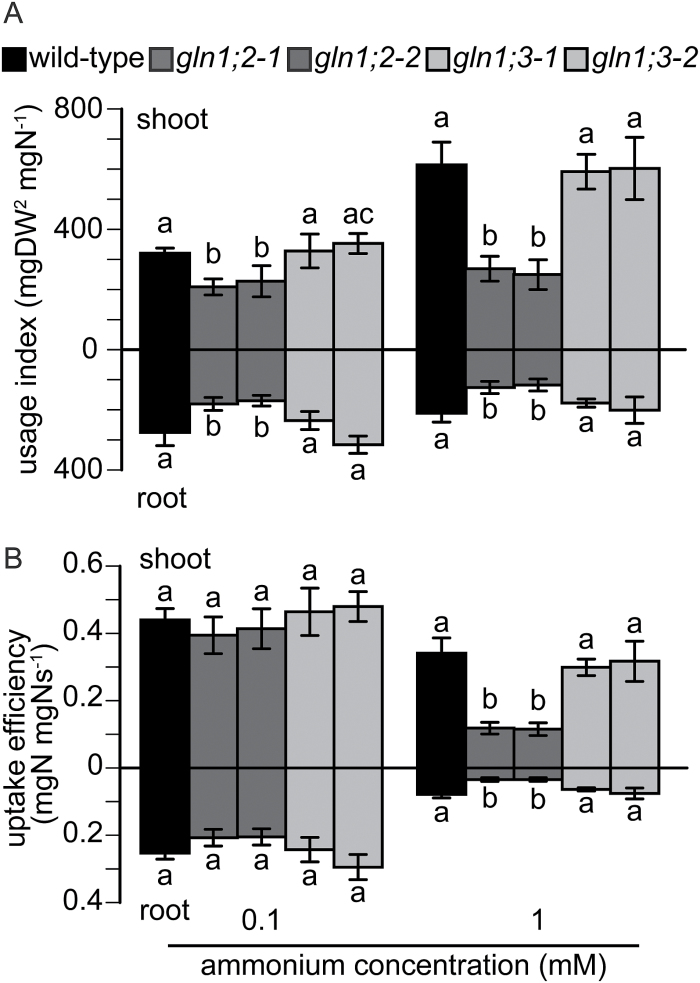
Nutrient use efficiency in roots and shoots of the wild-type (WT) and the *GLN1;2* and *GLN1;3* insertion lines. (A) Usage index (UI) was calculated as UI = Sw × (Sw/N), where Sw is shoot weight, and N is nitrogen in organs. (B) Uptake efficiency (UpE) was calculated as UpE = Nt/Ns where Nt is total nitrogen in the plant and Ns is nitrogen supply (g per plant). Data are means ±SD (*n* = 6) for the WT, and the *GLN1;2* and *GLN1;3* insertion lines (as indicated in the key). Plants were grown hydroponically for 6 weeks, and supplemented with either 0.1 or 1.0 mM ammonium, and 10 µM nitrate. One-way ANOVA followed by Bonferroni tests were used, and significant differences at *P*<0.05 within each group are indicated by different letters.

Figure 5 illustrates the UI and UpE in the *GLN1;2* and *GLN1;3* insertion lines compared with the WT. The effects of T-DNA insertion in *GLN1;2* on the UI and UpE were dramatic. The *GLN1;2* insertion lines showed a markedly reduced UI, especially under higher ammonium supply, whereas the *GLN1;3* insertion lines did not show changes in the UI under high or low ammonium supply in comparison with the WT (Fig. 5A). *GLN1;2* insertion reduced the UI by 30% under 0.1 mM ammonium and by 50% under 1 mM (Fig. 5A) in comparison with the WT. UpE was reduced in the *GLN1;2* insertion lines only at higher ammonium supply (Fig. 5B), with a 65% decrease in UpE under 1 mM ammonium. *GLN1;3* insertion did not change UpE under either high or low ammonium supply.

**Fig. 6.**
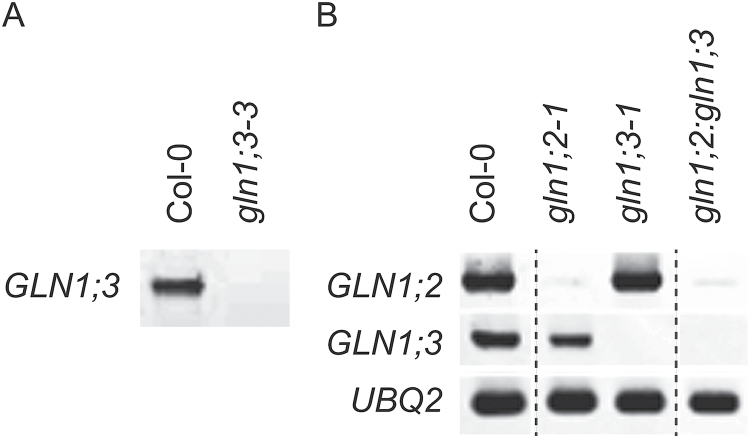
Isolation of the double-insertion line for *GLN1;2* and *GLN1;3*. (A) Reverse transcription polymerase chain reaction (RT-PCR) analysis of root RNA from the single-insertion line for *GLN1;3*. The *GLN1;3* insertion line named *gln1;3-3* is identical to GLN1;3 KO in a previous study by Dragićević *et al*. (2014). (B) RT-PCR analysis of root RNA from single-insertion lines and their corresponding wild-type and from the double-insertion line. Plants were grown hydroponically for 6 weeks, and supplemented with 0.1 mM ammonium and 10 µM nitrate as the major nitrogen source.

**Fig. 7.**
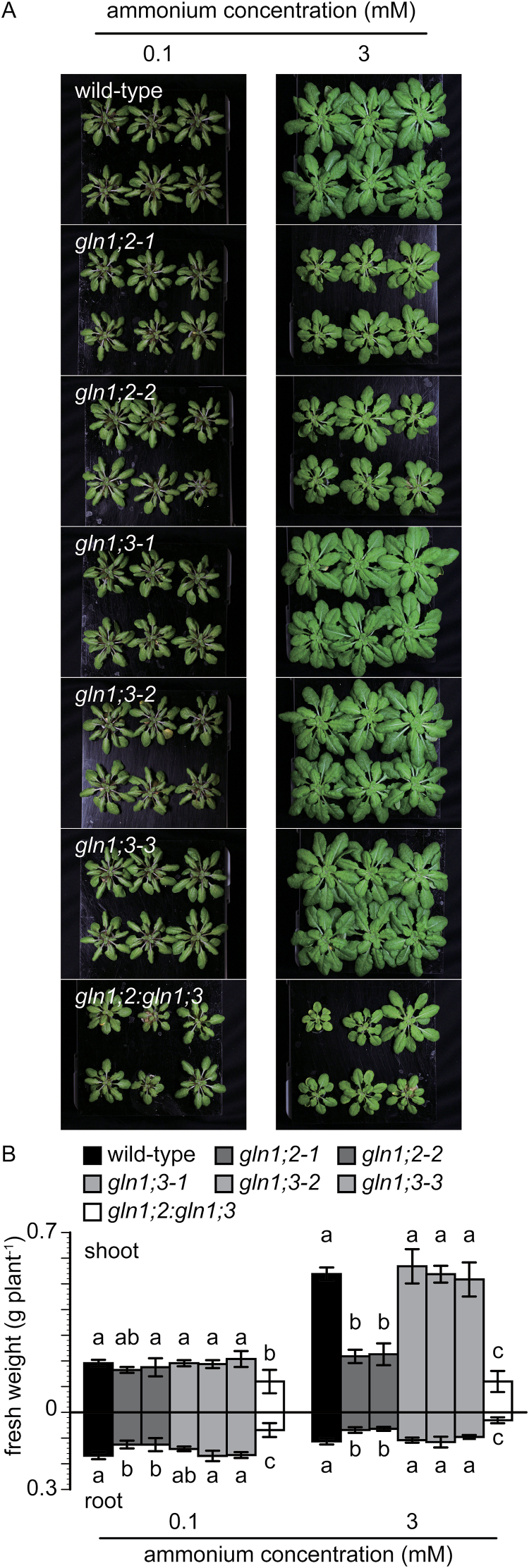
Growth of the wild-type (WT) and the *GLN1;2* and *GLN1;3* insertion lines under low nitrate supply, and the effect of ammonium supply in nutrient solution. (A) Phenotype of the WT and the insertion lines for *GLN1;2* and *GLN1;3*. (B) Shoot and root dry weights of the WT the *GLN1;2* insertion line, the *GLN1;3* insertion line, and the *GLN1;2:GLN1;3* double-insertion line (as indicated in the key). Plants were grown for 6 weeks in nutrient solutions containing 0.1 or 3 mM ammonium and 10 μM nitrate as the nitrogen source. Data are means ±SD (*n* = 6). One-way ANOVA followed by Bonferroni tests were used, and significant differences at *P*<0.05 within each group are indicated by different letters. (This figure is available in colour at *JXB* online.)

To clarify the overlapping functions of GLN1;2 and GLN1;3, two *gln1* insertion lines, *gln1;2* and *gln1;3*, were crossed, and a double-insertion line, *gln1;2:gln1;3* was isolated (Fig. 6). RT-PCR analysis indicated that the double-insertion line expressed neither *GLN1;2* nor *GLN1;3* (Fig. 6B). Statistical analysis of fresh weight is presented in Fig. 7. In the single-insertion *gln1;2*, the fresh weight was decreased by half under 3 mM ammonium conditions, whereas in the single-insertion *gln1;3*, it was not much different from the WT. The fresh weight of *gln1;2:gln1;3* was significantly different from the single-insertion lines. Under 0.1 mM ammonium conditions, it was decreased by 36% compared with *gln1;2*, and decreased by 46% compared with *gln1;3*. Under 3 mM ammonium conditions, it showed 48% and 77% reductions, respectively (Fig. 7).

**Fig. 8.**
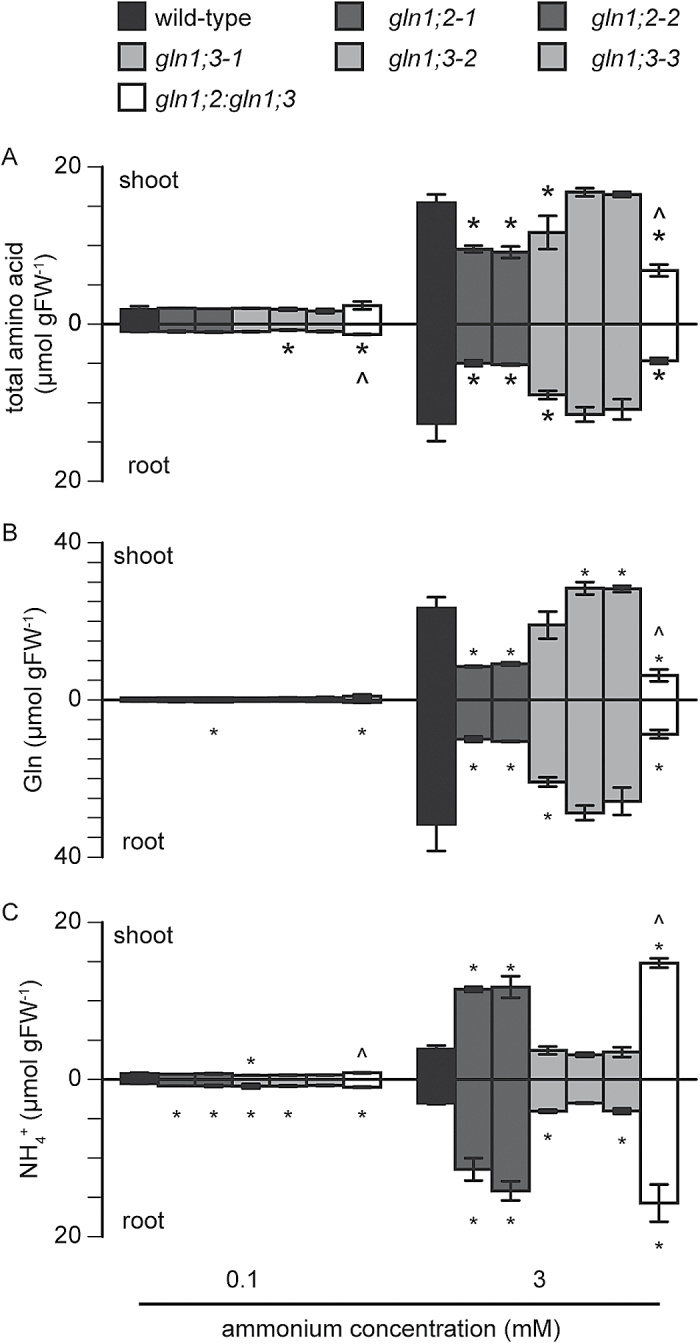
Ammonium accumulation and amino acid reduction in the *GLN1;2* and *GLN1;3* insertion lines under ammonium supply. The concentrations of total free amino acids (A), free glutamine (B), and free ammonium (C) were measured in roots and shoots of the wild-type (WT), the *GLN1;2* insertion line, the *GLN1;3* insertion line, and the *GLN1;2:GLN1;3* double-insertion line as indicated in the key). Plants were grown hydroponically for 6 weeks, and supplemented with 0.1 or 3 mM ammonium and 10 µM nitrate as the major nitrogen source. Data are means ±SD (*n* = 3). One-way ANOVA followed by Dunnett tests were used, and significant differences at *P*<0.05 between the WT and the *GLN1;2* or *GLN1;3* insertion lines are indicated with asterisks (*), and significant differences between the *GLN1;2* insertion line and the *GLN1;2:GLN1;3* double-insertion line are indicated with a circumflex (^).

To distinguish the functions of two root GS1 isozymes in ammonium assimilation in Arabidopsis, the free amino acid and ammonium concentrations were compared between the *GLN1* insertion lines and the WT under 0.1 and 3 mM ammonium supply. Figure 8 summarizes the changes in free ammonium and glutamine in the WT and *GLN1* insertion lines. Ammonium concentration was sharply increased in the *GLN1;2* insertion lines (Fig. 8C), whereas glutamine (Fig. 8B) as well as total amino acid (Fig. 8A) concentrations were decreased. Supplementary Fig. S3 shows the amino acid composition in shoots and roots of the WT and insertion lines. Glutamine accounted for >40% of the total amino acids in the shoots and >70% in the roots at 3 mM ammonium. Serine, asparagine, and arginine accounted for approximately 10% of the total amino acids in the shoots at 3 mM ammonium. A loss of GLN1;2 led to a decrease in the ratio of glutamine but an increase in the of ratio serine in the whole plant at 3 mM ammonium. The ratios of aspartate, threonine, and alanine were increased in the *GLN1;2* insertion lines.

**Fig. 9.**
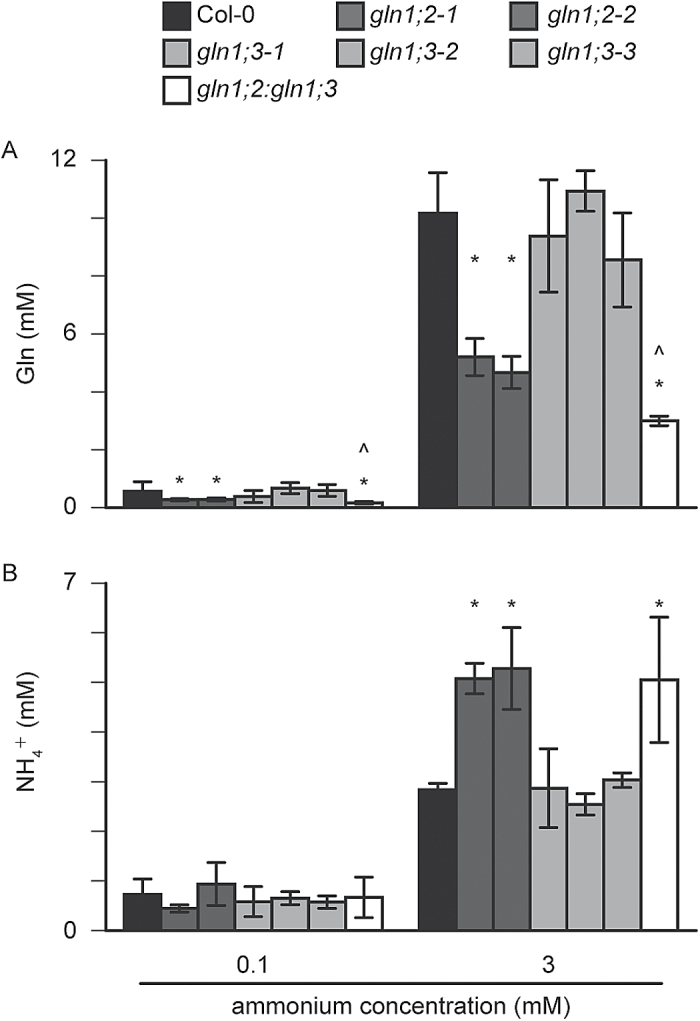
Ammonium accumulation and glutamine reduction in xylem sap of insertion lines after ammonium was supplied. (A) The concentration of glutamine in xylem sap of the wild-type (WT) and T-DNA insertion lines for *GLN1;2* and *GLN1;3*. (B) The concentration of ammonium in xylem sap of the WT, the *GLN1;2* and *GLN1;3* insertion lines, and the *GLN1;2:GLN1;3* double-insertion line (as indicated in the key). Plants were grown for 42 d in nutrient solution containing 2 mM ammonium nitrate and then transferred to nutrient solution without nitrogen. After 3 d, the plants were transferred again to a nutrient solution containing either 0.1 or 3 mM ammonium and 10 μM nitrate. After 24 h, plants were excised and xylem sap was collected. Data are means ±SD (*n* = 4). One-way ANOVA followed by Dunnett tests were used, and significant differences at *P*<0.05 between the WT and the *GLN1;2* or *GLN1;3* insertion lines are indicated with an asterisks (*) and significant differences between the *GLN1;2* insertion line and the *GLN1;2:GLN1;3* double-insertion line are indicated with a circumflex (^).

Given that the rice *GS1;2* mutant showed increased ammonium and decreased glutamine (Funayama *et al.*, 2013), we investigated the changes in these compounds in xylem exudates from the Arabidopsis *GLN1;2* and *GLN1;3* insertion lines after supplying ammonium. Figure 9 illustrates the changes in glutamine and ammonium concentrations in the xylem sap over 24 h after supplying ammonium. *GLN1;2* insertion lines resulted in a 50% decrease in glutamine in comparison to the WT (Fig. 9A). The ammonium concentration was higher in *GLN1;2* than in the WT (Fig. 9B).

### GLN1;2 absence reveals a function for GLN1;3 under ammonium nutrition

The *GLN1;3* insertion lines did not show reductions in dry weight under any conditions tested except for 0.5 mM ammonium supply (Fig. 3A, B), when root dry weight was decreased by 20–30% (Fig. 3B). Since variability was observed among plants (Fig. 3), the third insertion line, *gln1;3-3*, was used in further analysis (Fig. 6). RT-PCR showed no detectable signal for *GLN1;3* in the *gln1;3-3* insertion line (Fig. 6). Given that no significant differences between the WT and the *GLN1;3* insertion lines were observed (Figs 3–5, 7, 9, and Supplementary Fig. S2), *gln1;2* and the *gln1;2:gln1;3* double-insertion line (Fig. 6) were compared under 0.1 and 3 mM ammonium. The *GLN1;3* insertion lines showed no significant decrease in fresh weight under the tested conditions (Fig. 7). Compared with *gln1;2*, the root fresh weight was decreased by half and the shoot dry weight was decreased 30%–45% in *gln1;2:gln1;3*.

Given that *gln1;2:gln1;3* showed decreased biomass, free amino acids and ammonium were measured at the 0.1 and 3 mM ammonium conditions (Fig. 8). The *GLN1;3* insertion lines showed no clear changes in ammonium concentration (Fig. 8C). No significant differences were observed in the concentrations of total amino acids (Fig. 8A) and glutamine (Fig. 8B) between *gln1;3* and the WT. The total amino acids and glutamine in *gln1;2:gln1;3* were lower than those in the *gln1;2* shoots (Fig. 8A, B), whereas ammonium in the double-insertion line was higher than in *gln1;2* (Fig. 8C). Supplementary Fig. S3 shows that a loss of GLN1;3 did not dramatically change the amino acid composition.

Xylem sap analysis indicated that the glutamine concentration in *gln1;2:gln1;3* was significantly lower than that in *gln1;2* (Fig. 9A), whereas there was no significant difference in ammonium concentration (Fig. 9B). Under all the conditions tested, the *GLN1;3* insertion lines showed no statistical differences from the WT (Fig. 9).

### *The promoter activities of* GLN1;2 *are enhanced in the epidermis and cortex cell layers, and* GLN1;3 *is constitutively localized in the pericycle*

**Fig. 10.**
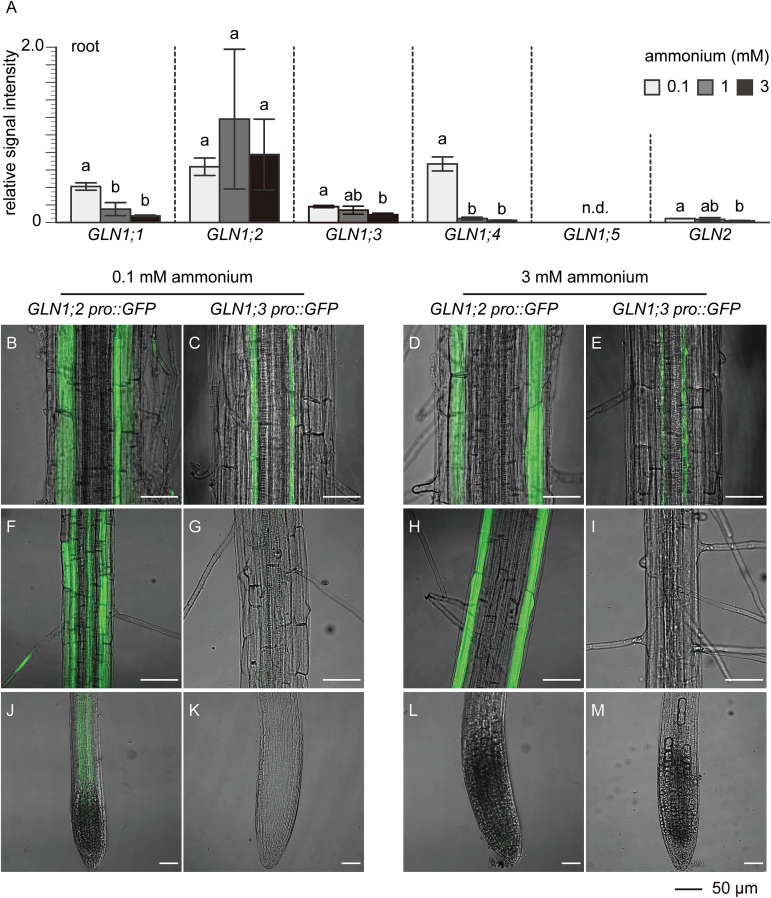
Organ and cell type-specific expression of *GLN1* genes in Arabidopsis roots. (A) Quantitative real-time polymerase chain reaction (qPCR) analysis of root RNA from the wild-type (WT) using gene-specific primers for *GLN1;1*, *GLN1;2*, *GLN1;3*, *GLN1;4*, *GLN1;5*, and *GLN2*. Plants were grown in nutrient solutions containing either 0.1, 1, or 3 mM ammonium (as indicated in the key) and 10 μM nitrate for 6 weeks. Ubiquitin2 (*UBQ2*) was used to standardize the signal intensity. Data are means ±SD (*n*=3 or 4). One-way ANOVA followed by Bonferroni tests were used, and significant differences at *P*<0.05 within each group are indicated by different letters. (B–M) Localization of the promoter activities of *GLN1;2* (B, D, F, H, J, L) and *GLN1;3* (C, E, G, I, K, M). Transgenic plants expressing either the *GLN1;2 promoter:GFP* or the *GLN1:3 promoter:GFP* fusion gene constructs were grown for 6 weeks in nutrient solutions containing 0.1 mM (B, C, F, G, J, K) or 3 mM (D, E, H, I, L, M) ammonium and 10 μM nitrate as the nitrogen source. Whole-mount images from root tips (J–M), root hair zones (F–I), and mature parts (B–E) were acquired by confocal laser scanning microscopy. Scale bars represent 50 μm.

Figure 10A summarizes the expression of *GLN* genes in Arabidopsis roots under 0.1, 1, and 3 mM ammonium supply. Roots greatly accumulated *GLN1;2* under both high and low ammonium supply. Other *GLN* genes, *GLN1;1*, *GLN1;3*, *GLN1;4*, and *GLN2*, were all more highly expressed at 0.1 mM ammonium than under higher-ammonium conditions (Fig. 10A). *GLN1;2* accounted for only 34% of the total *GLN* transcripts at 0.1 mM ammonium supply, but for almost 80% at 1 and 3 mM. *GLN1;5* was not detectable.

Figure 10B–M illustrates the localization of *GLN1;2* and *GLN1;3* promoter activity under 0.1 or 3 mM ammonium conditions. *GLN1;2* promoter activity was mainly localized in the epidermis and cortex (Fig. 10B, D, F, H), whereas *GLN1;3*-dependent GFP was localized mainly in the pericycle of mature roots (Fig. 10C, E). However, *GLN1;3* promoter activity was localized in neither the root hair zone (Fig. 10G, I), nor the root tips (Fig. 10K, M). Variable ammonium concentrations did not change the localization of *GLN1;3* promoter activity (Fig. 10). On a vertical agar culture, ammonium supply greatly induced *GLN1;2* promoter activity in the rhizosphere, whereas it did not change *GLN1;3* promoter activity (see Supplementary Fig. S4).

**Fig. 11.**
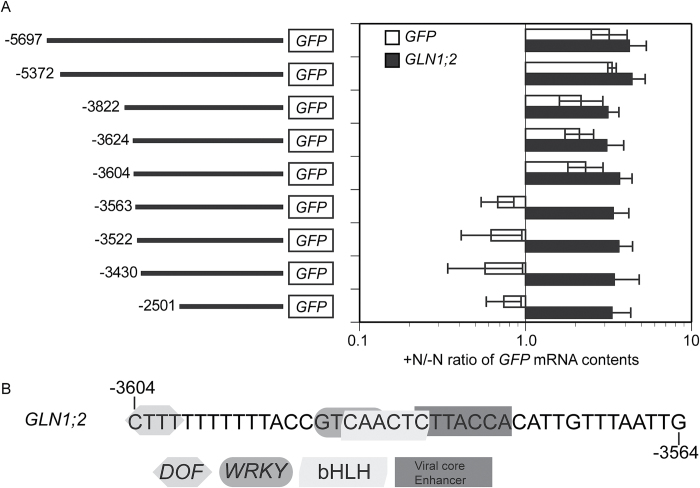
Deletion analysis of ammonium-responsive *GLN1;2* promoter in roots. (A) 5′ deletion analyses between −5697 and −2501 of the *GLN1;2* promoter were performed. Green fluorescent protein (*GFP*) was quantified in the roots of each transgenic plant with real time polymerase chain reaction (qPCR) using specific primers. At least five independent lines of T2 transformants from each construct were grown on MGRL medium for 2 weeks, after which plants were subjected to nitrogen starvation for 3 d prior to treatment, and then transferred to modified MGRL medium either without nitrogen (–N) or with 10 mM ammonium chloride (+N). Data are the ratio of +N and –N. Means of five to ten independent samples and the standard deviations are presented. (B) There are four predicted motifs for binding to Dof (−3604 CTTT −3601), WRKY proteins (−3590 GTCAA −3586), bHLH (−3588 CAACTC −3583), and viral core enhancer (−3583 CTTACCA −3577) in the 41-bp region.

To identify the regulatory region for the ammonium response of *GLN1;2* gene expression, we compared the responses of truncated versions of *GLN1;2* promoter-GFP constructs in transgenic Arabidopsis plants (Fig. 11 and Supplementary Fig. S5). The full-length promoter, containing a genomic region 5697 bp upstream of the *GLN1;2* translational start codon, responded to ammonium in the growth medium and this resulted in a significant increase in *GFP* mRNA accumulation (Fig. 11A). Quantitative real-time RT-PCR revealed that this full-length promoter could drive *GFP* expression on ammonium supply, cumulating in *GFP* levels up to three-fold those under the control nitrogen-starved conditions (Fig. 11A and Supplementary Fig. S5). The induction of *GFP* accumulation, driven by this full-length promoter, was consistent with the increased accumulation of *GLN1;2*. Following the 5′-deletion series of the *GLN1;2* promoter-GFP constructs, there was no great difference in the fold-change induction of *GFP* expression as far as the position −3604. However, truncation of the promoter to −3563 drastically reduced the *GFP* expression (Fig. 11A). Nevertheless, the endogenous *GLN1;2* responded to the ammonium supply.

## Discussion

Earlier studies showed that a small amount of supplied nitrate (Krouk *et al.*, 2006; Yuan *et al.*, 2007; Garnica *et al.*, 2010) or pre-culture in nitrate medium (Hachiya *et al.*, 2012; Sarasketa *et al.*, 2014) alleviated ammonium toxicity. Supplemented with a small amount of nitrate, the present study showed that ammonium toxicity appeared at 3 mM in hydroponic culture and that nitrogen deficiency appeared at 0.3 mM (Fig. 1). It was evident that the optimal ammonium concentration in the nutrient solution was 1 or 2 mM. The phenotypes observed below 3 mM ammonium were consistent with general ammonium assimilation but not with ammonium toxicity (see Supplementary Fig. S1).

Three independent T-DNA insertion lines for *GLN1;3* and *GLN1;2* (Fig. 2) and a double-insertion line for *GLN1;2* and *GLN1;3* were isolated (Fig. 6). The growth of the insertion lines was compared with that of the WT in hydroponic culture (Figs 1, 3, and 7). The contribution of *GLN1;3* to ammonium assimilation was not major in comparison with that of *GLN1;2* (Figs 3 and 7). The comparison of *gln1;2:gln1;3* with *gln1;2* indicated the small but significant contribution of GLN1;3 to ammonium assimilation in roots (Figs 6–9). GLN1;3 revealed its function only when GLN1;2 was not functional.

*GLN1;3* promoter activity was localized to the pericycle and was independent of the external ammonium concentration (Fig. 9). In the root, the pericycle is required for xylem loading and for lateral root initiation (Beeckman and De Smet, 2014). The pericycle-associated GLN1;3 might be involved in xylem loading of glutamine. Indeed, xylem sap glutamine in *gln1;2:gln1;3* was significantly lower than that in *gln1;2* (Fig. 9), suggesting that the loading of glutamine to the xylem was partly dependent on GLN1;3. The growth and localization might suggest that the GLN1;3 assimilates concentrated symplastic ammonium around the stele. These findings extend the function of GLN1;3 from enzymatic characteristics to physiological functions within the plant. A previous study showed that ammonium supply triggers lateral root development (Lima *et al.*, 2010). Future work should focus on the contribution of pericycle-localized GLN1;3 to root system architecture under ammonium supply.

It is likely that differences in spatial and temporal expression of *GLN1;2* and *GLN1;3* determine the different responses of these two *GLN1* insertion lines to various ammonium concentrations. However, the post-translational regulation of the two GS1 isozymes *in planta* remains unknown. Growth analysis of transgenic plants expressing *GLN1;3* driven by the *GLN1;2* promoter in a *GLN1;2* and *GLN1;3* double-insertion line may be a promising approach.

Previous studies localized *GLN1;2* promoter activity in the root vascular tissues (Ishiyama *et al.*, 2004; Lothier *et al.*, 2011; Guan *et al.*, 2015). In the present study, the *GLN1;2* promoter was longer than that in previous studies because the shorter promoter (Ishiyama *et al.*, 2004) did not respond to the ammonium supply (Fig. 10). The longer *GLN1;2* promoter-GFP showed the localization of GLN1;2 in the epidermis and cortex under ammonium supply (Figs 10, 11, and Supplementary Fig. S4). The promoter deletion analysis suggested that at least the sequences between −3604 and −3563 bp are necessary to enhance *GLN1;2* transcriptional activity in response to ammonium supply in the roots. A database search on plant cis-acting regulatory DNA elements (Higo *et al.*, 1999) showed that this region could be recognized by four types of transcriptional factors (Fig. 11B), namely DNA-binding with one finger (DOF) (Yanagisawa, 1996), WRKY, bHLH, and viral core enhancer. This is in good agreement with previous studies suggesting DOF-dependent nitrogen metabolism (Yanagisawa *et al.*, 2004) and DOF-dependent *GLN* expression (Rueda-López *et al.*, 2008). *GLN1;2* accumulation in response to ammonium supply initially occurs in the epidermis cell layers of Arabidopsis roots, where the enzyme would have major metabolic functions in assimilating the ammonium uptake from the rhizosphere.

In addition to localization studies, a reverse-genetic analysis also suggested the importance of GLN1;2 in ammonium assimilation in Arabidopsis (Lothier *et al.*, 2011; Guan *et al.*, 2016). Because the Casparian strip blocks apoplastic ammonium transport between pericycle cells and the soil solution (Loqué *et al.*, 2006), most apoplastic and symplastic ammonium should be assimilated by GLN1;2. GLN1;2 contributed to ammonium assimilation not only at higher concentrations of 2–20 mM, as shown in previous studies (Lothier *et al.*, 2011; Guan *et al.*, 2016) but also at lower concentrations of 0.3 mM (Fig. 3). Because ammonium in the soil solution varies from 0.1 to 0.8 mM (Miller *et al.*, 2007), the presence of such a broad GLN1;2 contribution is a realistic finding.

Ammonium supply increased the proportion of *GLN1;2* in the total *GLN* isogene pool (Fig. 10). This was consistent with results obtained in agar culture (Ishiyama *et al.*, 2004). Given that *GLN1;5* appears to be a pollen-specific GS1 (Schmid *et al.*, 2005; Soto *et al.*, 2010) and that it was not detectable in roots (Figs 2 and 10), the five *GLN* genes may reflect the population of root *GLN*. Increasing the ammonium concentration severely inhibited the growth of *gln1;2* (Figs 3 and 7). Inhibition of both nitrogen use and nitrogen acquisition (Fig. 5) resulted in reduced nitrogen concentration (Fig. 4). These results are partially consistent with those of previous studies (Lothier *et al.*, 2011). In addition to those phenotypes, *GLN1;2* insertion dramatically increased the free ammonium concentration not only in the plant organs but also in xylem exudate, whereas free glutamine concentration was decreased (Figs 8, 9, and Supplementary Fig. S3). Xylem sap analysis indicated that GLN1;2-dependent ammonium assimilation mainly occurred in roots when the ammonium concentration was <3 mM. Excess ammonium supply appears to saturate the capacity of root GLN1;2; therefore, shoot GLN1;2 is essential for overcoming ammonium toxicity (Guan *et al.*, 2016).

It is already known that ammonium supply triggers the accumulation of glutamine (Clark, 1936). Amino acid composition analysis showed that arginine accounted for approximately 15% in the shoots at 3 mM ammonium (see Supplementary Fig. S3), whereas arginine accounted for only <1% in nitrate-grown plants (Lothier *et al.*, 2011). Due to its having the highest nitrogen to carbon ratio among the 21 proteinogenic amino acids, arginine is a major storage for organic nitrogen in plants (Winter *et al.*, 2015). Accumulated glutamine appears to be converted to arginine in the shoots.

Neither the *GLN1;2* nor the *GLN1;3* insertion lines showed statistically different growth in either 1 mM or 10 mM nitrate supply in the present study. This result is not consistent with previous work (Lothier *et al.*, 2011), which showed a reduction in biomass in rosette leaves of the *GLN1;2* insertion line when plants were grown in 10 mM nitrate as the sole nitrogen supply, whereas there was no difference under the 2 mM nitrate supply. The reason for the mismatches between the two studies could be explained by the different culture conditions and genetic backgrounds used. There were differences in temperature, light, and nutrient (besides nitrate) concentrations. A previous study used the *GLN1;2* insertion line in Arabidopsis Ws as the genetic background, whereas the present work used Col. The growth of Ws and Col show differences under nitrate-supply conditions (Lothier *et al.*, 2011).

In conclusion, the contribution of GLN1;2, an ammonium-inducible GLN1, to ammonium assimilation was much higher than that of GLN1;3. GLN1;3 may assimilate the ammonium that is not assimilated by GLN1;2. Although the present study provides insights into the physiological functions of GLN1;2 and GLN1;3, they are not the only GLN1 isozymes expressed in the roots of Arabidopsis. It will be necessary to investigate the functions of GLN1;1 and GLN1;4 with high-affinity to ammonium to elucidate the full set of ammonium-assimilatory mechanisms in Arabidopsis plants.

## Supplementary Material

Supplementary Data

## Abbreviations:

GFP: green fluorescent protein
GOGAT: glutamate synthase
GS: glutamine synthetase
qPCR: quantitative real-time PCR
RT: reverse transcription
T-DNA: transfer DNA
UBQ: ubiquitin
UI: usage index
UpE: uptake efficiency
UPLC: ultra-performance liquid chromatography
