# Supplementary figures and images for: Contributions of two cytosolic glutamine synthetase isozymes to ammonium assimilation in Arabidopsis roots
Source: J Exp Bot. 2016 Dec 22;68(3):613–25. doi: 10.1093/jxb/erw454 (PMC5441914; doi:10.1093/jxb/erw454)

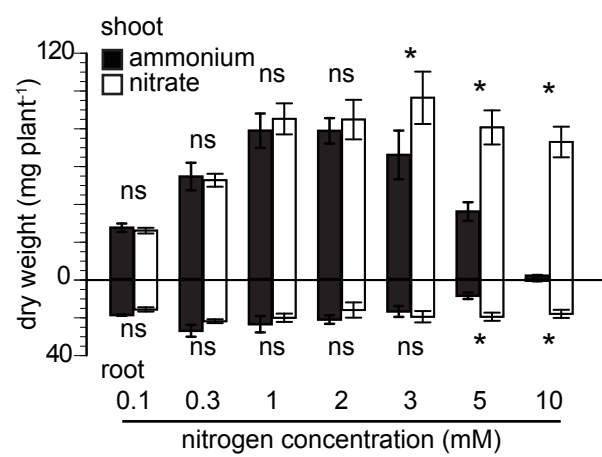

Fig. S1 Konishi et al

Supplement: Supplementary Data [file erw454_Supplementary_Data.zip › supplementary_figures_S1.pdf]

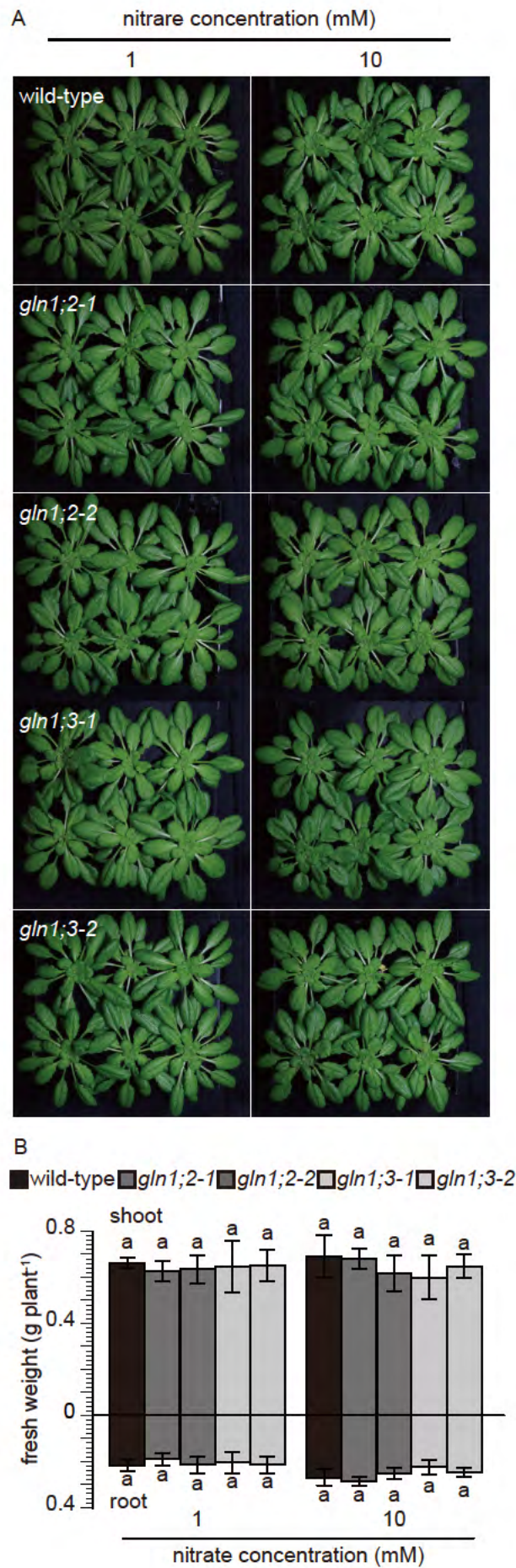

Fig. S2 Konishi et al

Supplement: Supplementary Data [file erw454_Supplementary_Data.zip › supplementary_figures_S2.pdf]

■ wild-type ■ *gln1;2-1* ■ *gln1;2-2* ■ *gln1;3-1* ■ *gln1;3-2* ■ *gln1;3-3* □ *gln1;2:gln1;3*

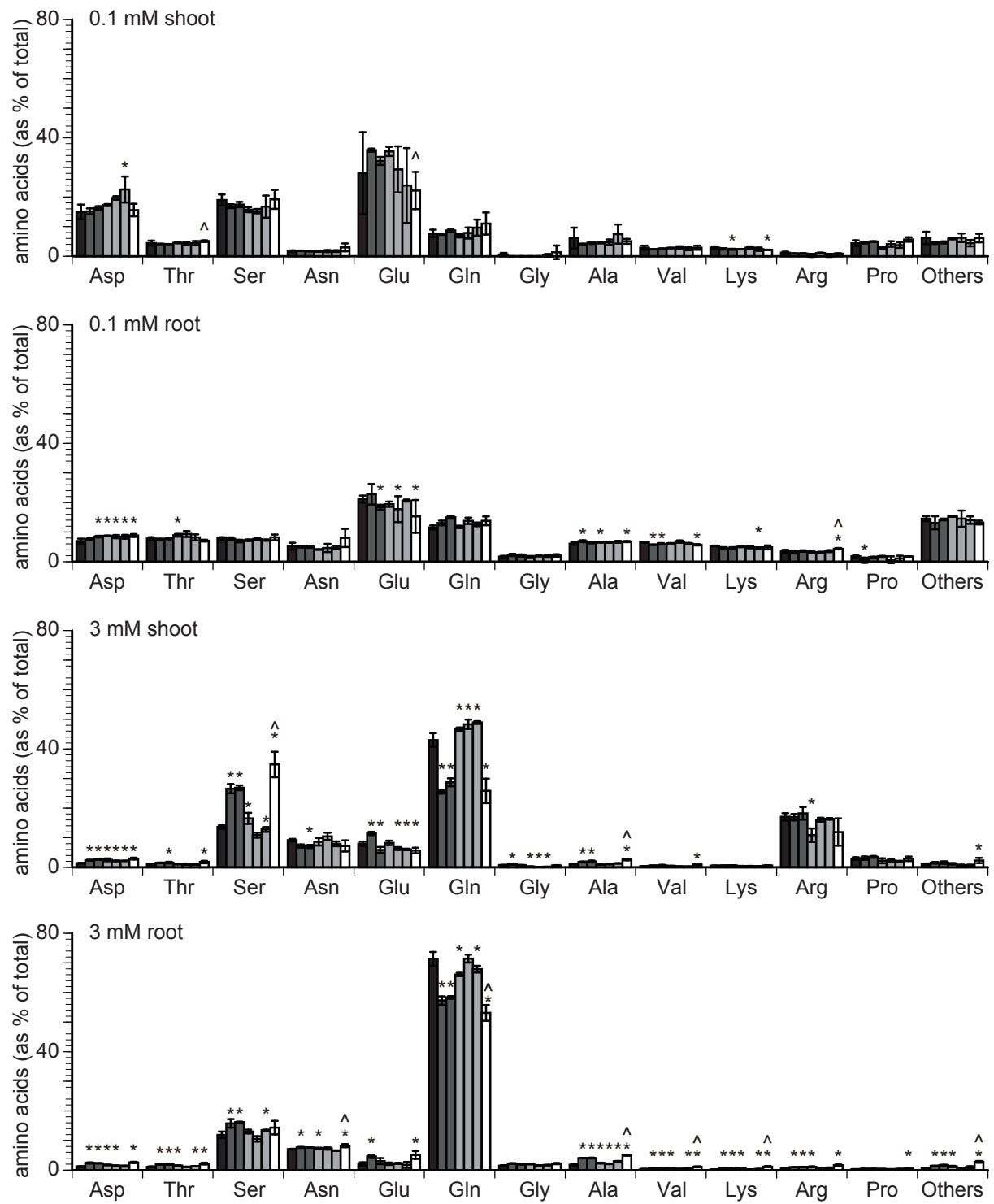

Fig. S3 Konishi et al

Supplement: Supplementary Data [file erw454_Supplementary_Data.zip › supplementary_figures_S3.pdf]

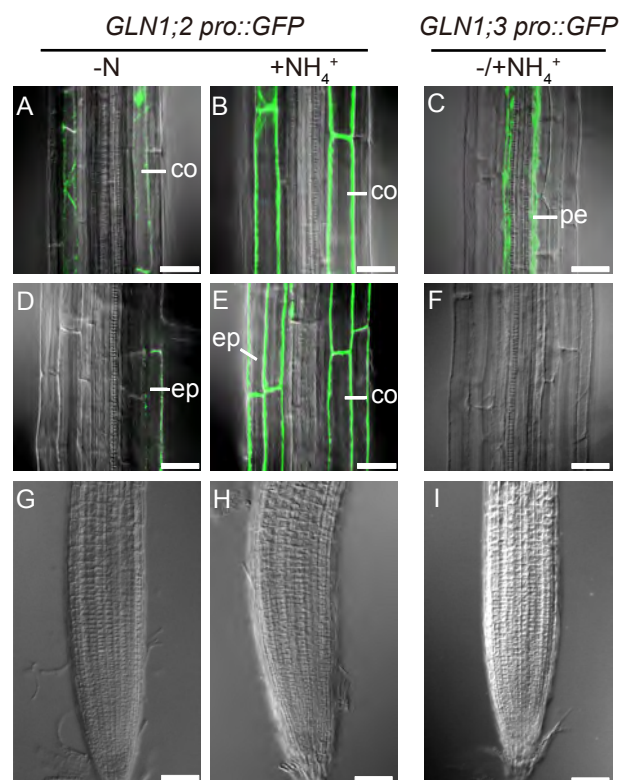

Fig. S4 Konishi et al

Supplement: Supplementary Data [file erw454_Supplementary_Data.zip › supplementary_figures_S4.pdf]

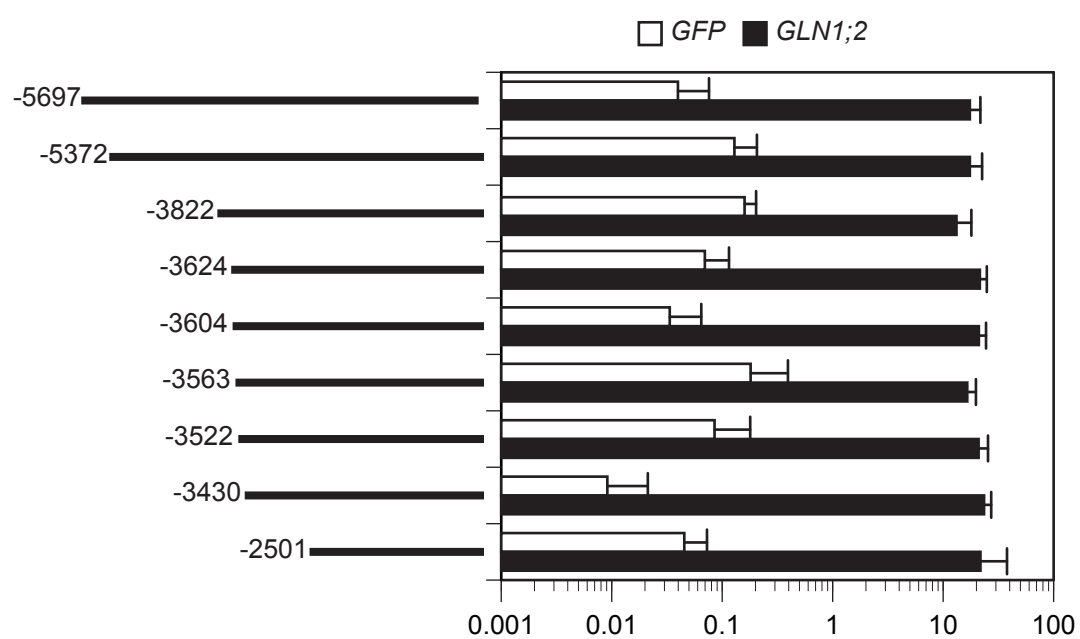

Fig. S5 Konishi et al

Supplement: Supplementary Data [file erw454_Supplementary_Data.zip › supplementary_figures_S5.pdf]
